# Lipidomic landscape of lipokines in adipose tissue derived extracellular vesicles

**DOI:** 10.3389/fmolb.2023.1281244

**Published:** 2023-10-31

**Authors:** Yan Zhang, Tingyan Dong, Muyao Wang

**Affiliations:** ^1^ Department of Oral and Maxillofacial Surgery, Tianjin Stomatological Hospital, School of Medicine, Nankai University, Tianjin, China; ^2^ Key Laboratory of Bioactive Materials (Ministry of Education), State Key Laboratory of Medicinal Chemical Biology, College of Life Sciences, Nankai University, Tianjin, China; ^3^ Tianjin Key Laboratory of Oral and Maxillofacial Function Reconstruction, Tianjin, China; ^4^ Department of Periodontology, Hexi Subsidiary Clinical-service of Tianjin Stomatological Hospital, The Affiliated Stomatological Hospital of Nankai University, Tianjin, China

**Keywords:** extracellular vesicles, lipokines, adipose tissue, obesity, lipidome

## Abstract

**Introduction:** Adipose tissue-derived extracellular vesicles (EVs-AT) are recognized as critical mediators of metabolic alterations in obesity-related diseases. However, few studies have focused on the role of lipids within EVs-AT in the development of obesity-related diseases.

**Methods:** In this study, we performed a targeted lipidomic analysis to compare the lipidome of EVs secreted by inguinal white adipose tissue (EVs-iWAT), epididymal white adipose tissue (EVs-eWAT), and interscapular brown adipose tissue (EVs-BAT) in lean and obese mice.

**Results:** We uncovered a comprehensive lipidomic map, revealing the diversity and specific lipid sorting in EVs-iWAT, EVs-eWAT, and EVs-BAT in obesity. Biological function analyses suggested that lipids encapsulated within EVs-AT of obese individuals might correlate with metabolism, pro-inflammatory response, and insulin resistance. These effects were particularly pronounced in EVs-eWAT and EVs-BAT.

**Conclusion:** Our findings indicated that EVs-AT serves as novel carriers for lipokines, thereby mediating the biological functions of EVs-AT. This study holds promise for the identification of new biomarkers for obesity-related diseases and the development of new strategies to combat metabolic diseases.

## Graphical Abstract

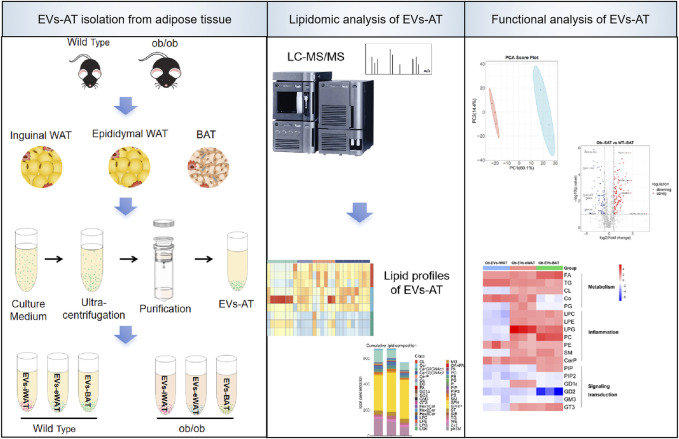

## 1 Introduction

The prevalence of obesity has increased substantially over the past four decades, imposing an enormous burden on public health (Kusminski et al., 2016). Obesity leads to chronic and systemic inflammation, contributing to various complications, including insulin resistance (IR), diabetes, non-alcoholic fatty liver disease (NAFLD), cardiovascular disease, and nephropathy (Reilly and Saltiel, 2017; Piché et al., 2020). The expansion of adipose tissue during obesity is a determinant of metabolic outcomes, prompting significant interest in adipose tissue biology (Sake et al., 2022).

Adipose tissue is commonly categorized into white, beige, and brown adipose tissues, each possessing distinct functions and phenotypes. White adipose tissue (WAT) primarily serves as an energy reservoir in the form of triglycerides. WAT is distributed across various depots and is primarily classified as subcutaneous WAT (sWAT) and visceral WAT (vWAT). The distribution and morphology of adipose tissue play a key role in determining the extent of adverse metabolic effects. Previous studies have demonstrated that sWAT exerts a protective effect on energy homeostasis (Marcelin et al., 2022). However, the dysregulation of vWAT is associated with increased risks of metabolic diseases (Cypess, 2022). Brown adipose tissue (BAT) is responsible for thermogenesis, and increasing BAT mass has been regarded as a promising strategy to combat obesity and related diseases. However, a recent study has shown that BAT from obese and hyperglycemic mice exhibits higher levels of inflammation (macrophages and T cell infiltration), endoplasmic reticulum (ER) stress, oxidative damage, and enhanced mitochondrial respiration activity (Villarroya et al., 2018; Carpentier et al., 2023). These studies highlight the involvement of various adipose tissue types in the development of systemic inflammation.

Adipose tissue secretes a multitude of protein factors, known as adipokines, to regulate the distal target organs (Scheja and Heeren, 2019). These adipokines have pro-inflammatory or anti-inflammatory properties, contributing to the development of systemic inflammation (Ouchi et al., 2011). Beyond polypeptide adipokines, the adipose tissue secretome comprises several chemically distinct classes of bioactive molecules, including bioactive lipids (Cao et al., 2008). These lipids are actively secreted into circulation and are termed as “lipokines” (Li et al., 2020). Recent studies have proposed lipokines to function as endocrine mediators facilitating cross-talk between adipose tissue and distant organs, including the pancreas, liver, and muscle, through diverse cell-surface and intracellular mechanisms of action (Li et al., 2020). For instance, obese adipose tissue secretes lipokines that induce inflammation in preadipocytes through a combined paracrine/autocrine manner, involving TLR4 and increased ROS, thus creating a paracrine loop that facilitates the differentiation of adipocytes with a proinflammatory profile (Renovato-Martins et al., 2020).

Extracellular vesicles (EVs) have emerged as a novel means of adipokine release and intercellular communication. However, few studies have focused on the endocrine roles of lipids within EVs-AT. Previous studies have reported that adipose tissue from lean mice releases ∼1% of its lipid content per day *via* EVs, a rate that more than doubles in obese animals (Flaher et al., 2019). Adipocytes release lipid-laden exosomes, delivering TAG locally to macrophages, thereby influencing their differentiation (Antonyak et al., 2019; Flaher et al., 2019). This indicates that lipokines are not only transported by EVs but also play vital roles in regulating EV-mediated functions. EVs provide a novel way for lipids to transfer and carry various lipid types (not limited to the secreted lipids). Therefore, characterization and quantification of the lipids in the EVs-AT will not only expand the list of lipokines but also provide insights into the health status of adipose tissue and the development of obesity and related diseases. To this purpose, we performed a targeted lipidomic analysis to compare the lipidome of EVs secreted by inguinal white adipose tissue (EVs-iWAT), epididymal white adipose tissue (EVs-eWAT), and interscapular brown adipose tissue (EVs-BAT) in lean and obese mice. Our work presents a comprehensive lipidomic landscape, revealing the lipid diversity and their specific sorting in EVs-iWAT, EVs-eWAT, and EVs-BAT. We also identified distinct EVs-AT lipid classes or species closely related to the obese state. Particularly, enrichment in some EV lipid species may represent novel biomarker candidates or mediators in metabolic dysfunctions associated with obesity.

## 2 Material and methods

### 2.1 Animals

The study was approved by the Research Ethics Committee of Tianjin Stomatological Hospital. Male 10-week-old wild type (WT) C57BL/6 mice and male 10-week-old obese C57BL/6 (ob/ob) mice were purchased from Nanjing Junke Bioengineering Co., Ltd. Animals were housed in a plexiglass cage (5 per cage) at a temperature (22ºC ± 3°C) and humidity (55% ± 15%). Animals were provided with food and sterile water *ad libitum* and kept on a 12-h light-dark cycle acclimated for 1 week before the study.

### 2.2 Isolation of adipose tissue-derived EVs

For this study, 5 g of adipose tissue (iWAT, eWAT, BAT) were separately collected from 8-week-old male wild-type or obese C57BL/6 mice. The tissue samples were washed extensively with sterile phosphate-buffered saline (PBS) to remove the debris and red blood cells. The tissue was finely sectioned into small pieces (1–2 mm (Piché et al., 2020)) under aseptic conditions. These explants were evenly distributed with a 5 mm spacing in three T75 flasks. After the explants adhered to the flasks (2 h), 10 mL *a*-MEM (without FBS) was gently added to each flask without disturbing the explants. The culture medium was collected after 24 h. Subsequently, the medium was centrifuged at 300 g for 10min, 2,000 g for 20 min, and filtered (0.22 μm filter) to remove the debris and large vesicles of cells. The resulting supernatant was further centrifuged at 100,000 g for 120 min 1 mL of the supernatant at the bottom of the ultracentrifuge tube was transferred to a sterile, and EVs isolation was performed using an exoEasy Maxi kit (QIAGEN) according to the manufacturer’s protocol. Briefly, 1 mL of the supernatant was mixed with an equal volume of “buffer XBP” and centrifuged at 500 g for 1 min at room temperature (RT) in the exoEasy spin column. Subsequently, the filter was washed with “buffer XWP” and the EVs were eluted in 400 µL elution “buffer XE”. The mix was then centrifuged at 500 g for 5 min at RT, reapplied to the filter, and centrifuged at 3,000 g for 5 min at RT. All samples were adjusted to a final volume of 500 µL with elution buffer and stored at −80°C (Supplementary Figure S1).

### 2.3 Extraction of EVs-AT lipids

Lipids were extracted according to the methyl-tert-butyl ether (MTBE) method. Briefly, the samples were spiked with an appropriate amount of internal lipid standards and then homogenized with 200 µL water and 240 µL methanol. Subsequently, 800 µL of MTBE was added, and the mixture was subjected to 20 min of ultrasound at 4°C followed by 30 min of settling at RT. The solution was then centrifuged at 14,000 g for 15 min at 10°C, yielding an upper organic solvent layer, which was collected and dried under nitrogen.

### 2.4 LC-MS/MS analysis

Reverse-phase chromatography was employed for LC separation using CSH C18 column (1.7 µm, 2.1 mm × 100 mm, Waters). The lipid extracts were re-dissolved in 200 µL 90% isopropanol/acetonitrile and centrifuged at 14,000 g for 15 min. Solvent A consisted of acetonitrile-water (6:4, v/v) with 0.1% formic acid and 0.1 Mm ammonium formate, while solvent B comprised acetonitrile isopropanol (1:9, v/v) with 0.1% formic acid and 0.1 Mm ammonium formate. The initial mobile phase was 30% solvent B at a flow rate of 300 μL/min, maintained for 2 min. Subsequently, solvent B was linearly increased to 100% in 23 min, followed by equilibration at 5% solvent B for 10 min. Mass spectra were acquired using Q-Exactive Plus in both positive and negative modes. ESI parameters were optimized and preset for all measurements as follows: Source temperature at 300°C, capillary temperature at 350°C, ion spray voltage at 3000V, S-Lens RF Level at 50%, and a scan range of m/z 200.

### 2.5 Data processing

Lipid species were identified using LipidSearch software version 4.2 (Thermo Scientific™) to process the raw data, peak alignment, retention time correction, and peak area extraction. LipidSearch contains data on more than 30 lipid classes, with information on more than 1,700,000 ion fragments. Adducts of +H and +NH4 were selected for positive mode searches, while -H and +CH3COO were selected for negative mode searches since ammonium acetate was used in the mobile phases. In the data extracted from LipidSearch, ion peaks with more than 50% missing values were removed from the group. After normalization and integration using the Perato scaling method, the processed data were imported into SIMPCA-P 16.1 (Umetrics, Umea, Sweden) for multivariate statistical analysis, including principal component analysis (PCA), partial least squares discriminant analysis (PLS-DA), and orthogonal partial least squares discriminant analysis (OPLS-DA). Lipids with significant differences were identified based on a combination of statistically significant thresholds of variable influence on projection (VIP>1) values obtained from the OPLS-DA model (multi-dimensional statistical analysis) and two-tailed Student’s t-test (*p*-value <0.05) applied to the raw data (unidimensional statistical analysis) using PASS 16 (https://www.ncss.com/software/pass/) before experiments.

### 2.6 Bioinformatics analysis

LINT-web (http://www.lintwebomics.info/single) is a web-based tool designed for comprehensive lipidomic dataset analysis (Li et al., 2021). The entire database was uploaded in LINT, and results of the lipid classification information, acyl-chain length analysis, and principal component analysis were exported as various plots, including stacked histograms of cumulative lipid classes, heatmaps, or volcano plots of lipid differences in EVs-iWAT, EVs-eWAT, EVs-BAT, and PCA score plots. Enrichment analysis for biological pathways was performed using LipidSig (http://chenglab.cmu.edu.tw/lipidsig) (Lin et al., 2021). The statistical significance of enrichment was assessed using the corrected *p*-value using the Benjamini–Hochberg method.

### 2.7 Statistical analysis

Each experiment was repeated at least three times. Data were expressed as mean values ± SD. Statistical analysis was performed with Student’s paired *t*-test. *p* < 0.05 was considered as statistically significant.

## 3 Results

### 3.1 Characteristics of adipose tissue-derived EVs (EVs-AT)

**FIGURE 1:**
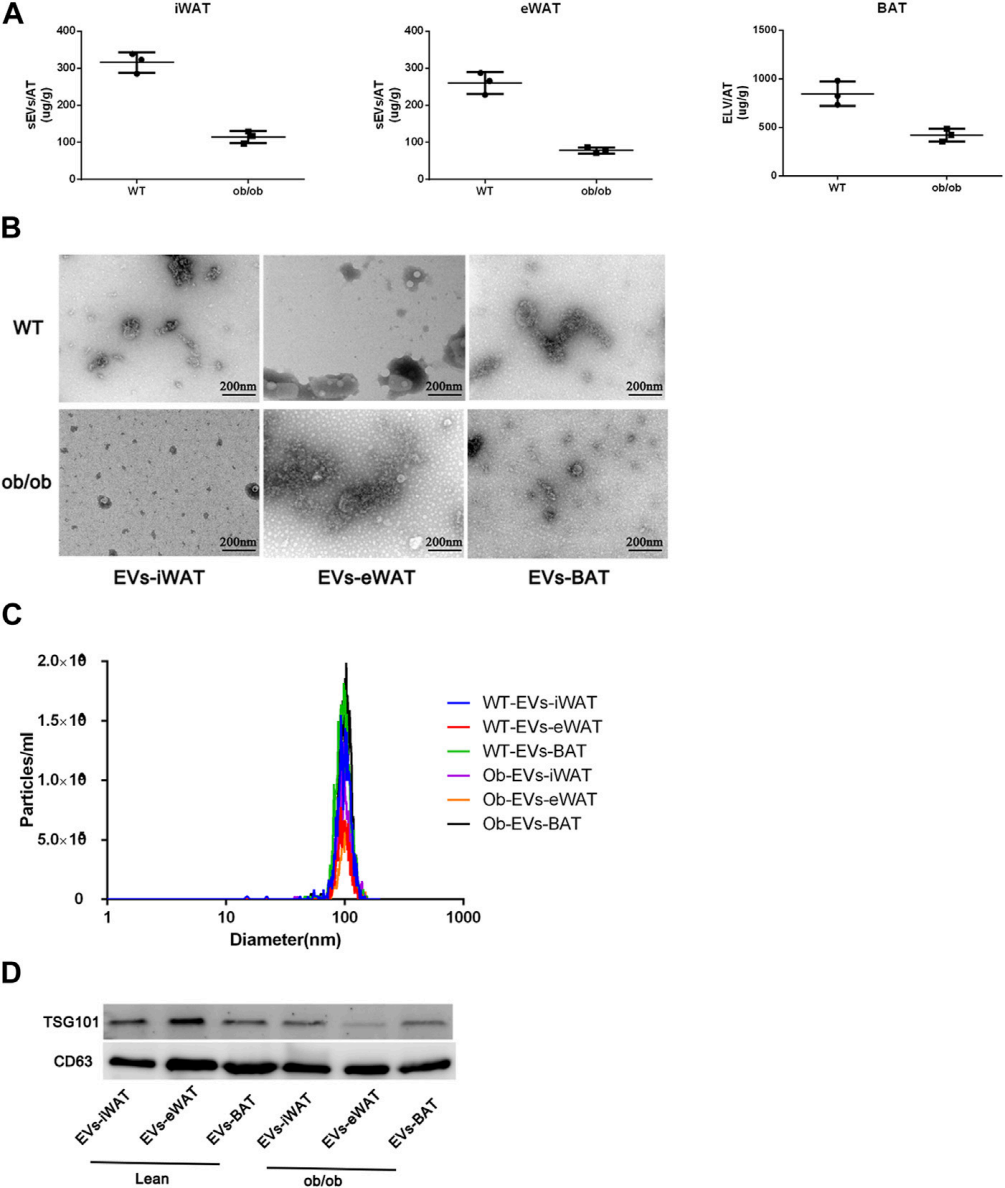
Characterization of EVs isolated from lean (wild type, WT) or obese iWAT (EVs-iWAT), eWAT (EVs-eWAT) and BAT (EVs-BAT). **(A)** Quantification analysis of EVs-AT in the lean or obese mice, Data represent mean ± SEM, **p < 0.01, Student’s t-test (Zhang et al., 2020); **(B)** Electron micrograph of EVs-iWAT, EVs-eWAT and EVs-BAT. Scale bar: 200 nm; **(C)** The size distribution of EVs-iWAT, EVs-eWAT and EVs-BAT as determined by NTA **(D)** Protein levels of CD63, TSG101 in EVs-iWAT, EVs-iWAT and EVs-BAT were detected by Western blot.

Firstly, we conducted a comprehensive characterization of EVs isolated from both lean and obese iWAT (EVs-iWAT), eWAT (EVs-eWAT), and BAT (EVs-BAT) using transmission electron microscopy (TEM), nanoparticle tracking analysis (NTA), and Western blot (WB) techniques. We found that obesity decreased the secretion of sEVs in all three adipose tissue types (Figure 1A). These vesicles exhibited a cup-shaped morphology (Figure 1B) with a diameter ranging from 50 to 200 nm (Figure 1C). The WB analysis demonstrated that the isolated EVs carried multiple positive EV markers, including CD63 and TSG101 (Figure 1D). The combined results from NTA, WB, and TEM analyses indicated no obvious differences in vesicle size distribution or appearance between lean or obese EVs-AT.

### 3.2 Lipidome analysis of normal/lean EVs-AT

**FIGURE 2:**
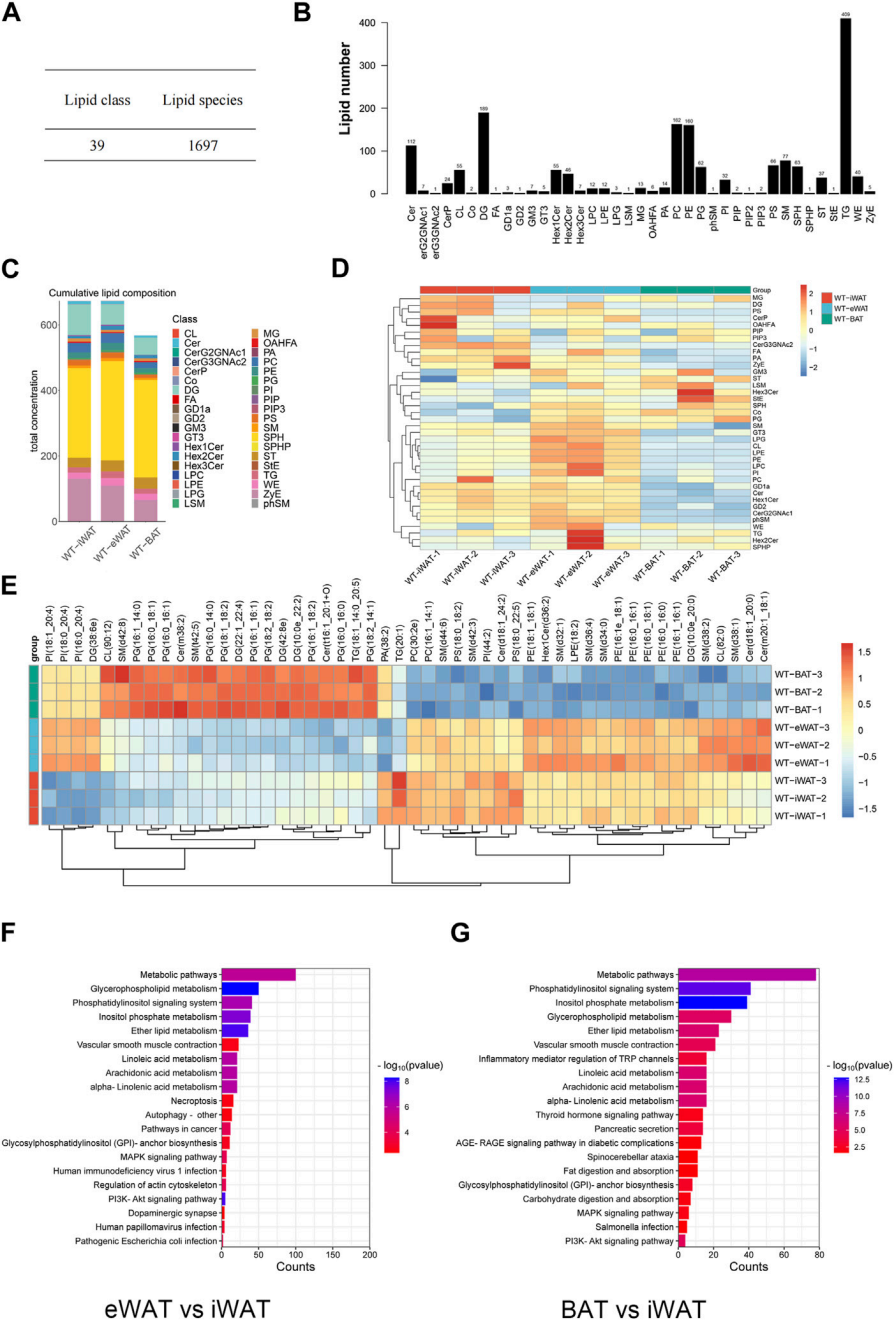
Lipidome analysis of normal/lean EVs-AT. **(A)** Total lipid molecules and lipid subclasses identified in EVs-AT; **(B)** Statistical map of lipid subclasses and lipid molecules; **(C)** A stacked histogram of cumulative lipid classes in EVs-iWAT, EVs-eWAT and EVs-BAT; **(D)** Heatmaps of lipid classes differences in EVs-iWAT, EVs-eWAT and EVs-BAT; **(E)** Hierarchical clustered heatmaps of lipid species in EVs-iWAT, EVs-eWAT and EVs-BAT; **(F)** KEGG analysis of the enriched lipid classes in EVs-eWAT compared with EVs-iWAT; **(G)** KEGG analysis of the enriched lipid classes in EVs-BAT compared with EVs-iWAT.

We conducted an in-depth absolute-quantitative mass spectrometric analysis to investigate the differences in lipid composition in EVs-iWAT, EVs-eWAT, and EVs-BAT. We identified 1,697 distinct lipid molecules from adipose tissue-derived EVs (AT-EVs), further classified into 39 lipid classes (Figure 2A; Supplementary Tables S1–S3). Notably, several lipid classes, including triglycerides (TG), diacylglycerols (DG), phosphatidylcholines (PC), phosphatidylethanolamines (PE), ceramides (CER), Sphingomyelin (SM) (Table 1) were the most abundant lipid types in AT-EV (Figure 2B). Furthermore, we observed specific enrichment of zymosterol (ZyE), wax ester (WE), and sterol lipids (ST) in AT-EVs when compared to the ExoCarta database (http://www.exocarta.org/). Importantly, AT-EVs were not abundant in cholesterol ester (CE) lipids, indicating limited lipoprotein contamination and validating the robustness of our EV isolation methodology from tissue. However, variations in lipid class concentrations were evident among EVs-iWAT, EVs-eWAT, and EVs-BAT (Figure 2C).

**TABLE 1:** Lipids abbreviation.

Cer	Ceramides
CerG2GNAc1	Simple Glc series
CerG3GNAc2	Simple Glc series
CerP	Ceramides phosphate
CL	Cardiolipin
Co	Coenzyme
DG	Diglyceride
FA	Fatty acid
GD1a	Gangliosides
GD2	Gangliosides
GM3	Gangliosides
GT3	Gangliosides
Hex1Cer	Hexosylceramide
Hex2Cer	Hexosylceramide
Hex3Cer	Hexosylceramide
LPC	Lyso-phosphatidylcholine
LPE	Lyso-phosphatidylethanolamine
LPG	Lyso-phosphatidylglycerol
LSM	Lyso-phosphatidylglycerol
MG	Diglyceride
OAHFA	(O-acyl)-1-hydroxy fatty acid
PA	Phosphatidic acid
PC	Phosphatidylcholine
PE	Phosphatidylethanolamine
PG	Phosphatidylglycerol
phSM	Sphingomyelin(phytosphingosine)
PI	Phosphatidylinositol
PIP	Phosphatidylinositol-3-trisphosphate
PIP2	Phosphatidylinositol-4,5-bisphosphate
PIP3	Phosphatidylinositol 3, 4, 5 trisphosphate
PS	Phosphatidylglycerol
SM	Sphingomyelin
SPH	Sphingosines
SPHP	Sphingoshine phosphate
ST	Sterol Lipids
StE	Stigmasteryl ester
TG	Triglyceride
WE	Wax ester
ZyE	Zymosteryl

We observed a significantly higher total lipid concentration in EVs-iWAT and EVs-eWAT than in EVs-BAT. Furthermore, EVs-iWAT, EVs-eWAT, and EVs-BAT exhibited a similar lipid pattern. In Figure 1D, a bar chart was presented to highlight visual differences in lipid variance between EVs-iWAT, EVs-eWAT, and EVs-BAT. WAT-derived EVs were enriched in Cer, FA, Hex1Cer, ZyE, GD1a, and GD2 compared to EVs-BAT (Supplementary Figure S2A). However, several glycerophospholipids classes, such as LPC, LPE, LPG, PI, and CL, were higher in EVs-eWAT than in EVs-iWAT (Supplementary Figure S2B). Conversely, CerG3GNAc2 were specifically enriched in EVs-iWAT (Supplementary Figure S2C), while PG and Co were enriched in Evs-BAT relative to EVs-eWAT (Supplementary Figure S2D).

Our lipidomic analysis was next extended to the individual molecular species of the identified lipid subclasses. Specifically, we identified ten PG species, Cer(m38:2), sm(t42:5) as enriched lipids and six PE species and seven SM species as less abundant lipids in EVs-BAT compared with EVs-iWAT and EVs-eWAT (Figure 2D). In contrast, six PE species and five SM species were specifically enriched in EVs-eWAT. Subsequent enrichment analysis for the differential lipids revealed that the metabolism, PI3K-AKT, MAPK signaling, and autophagy pathways were the main KEGG pathways in EVs-eWAT (Figure 2E), while EVs-BAT demonstrated enrichment in metabolism and cancer-related pathways (Figure 2F). Collectively, these findings highlighted the variance in lipidomic profiles and biological functions in EVs derived from distinct adipose tissue types.

### 3.3 Obesity alters EVs-AT lipidome

**FIGURE 3:**
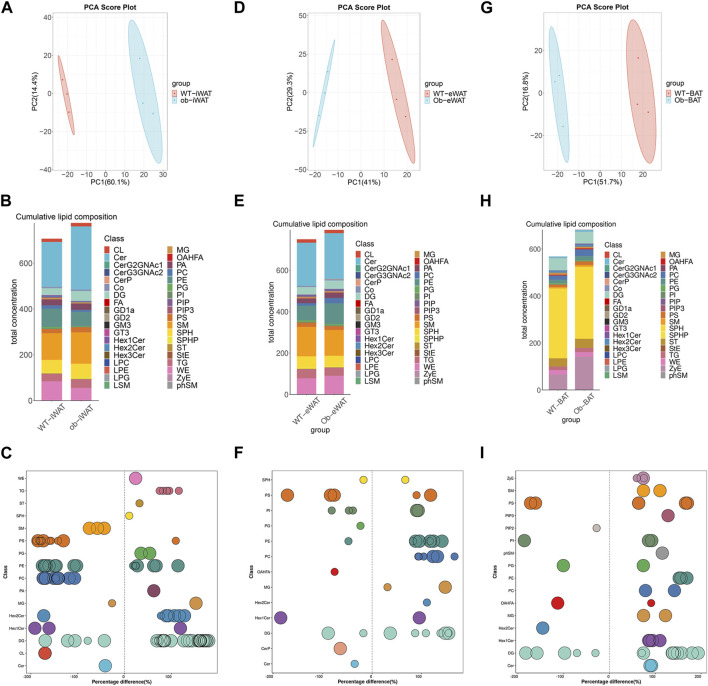
Obesity alters EVs-AT lipidome. **(A)** Principal component analysis (PCA) of EVs-iWAT lipidomes in lean and obese mice; **(B)** A stacked histogram of cumulative lipid classes in lean or obese EVs-iWAT; **(C)** The relative percentage difference in concentration of all quantified VIP lipid species between lean (left panel) or obese EVs-iWAT (right panel), each dot represents a lipid species, and the dot size indicates significance; **(D)** PCA of EVs-eWAT lipidomes in lean and obese mice; **(E)** A stacked histogram of cumulative lipid classes in lean or obese EVs-eWAT; **(F)** The relative percentage difference in concentration of all quantified VIP lipid species between lean (left panel) or obese EVs-eWAT (right panel), each dot represents a lipid species, and the dot size indicates significance; **(G)** PCA of EVs-BAT lipidomes in lean and obese mice; **(H)** A stacked histogram of cumulative lipid classes in lean or obese EVs-BAT; **(I)** The relative percentage difference in concentration of all quantified VIP lipid species between lean (left panel) or obese EVs-BAT (right panel), each dot represents a lipid species, and the dot size indicates significance.

To elucidate the effects of obesity on the lipid composition of EVs-AT, we performed a comparative lipidomic analysis between the obese and lean state on the leptin-deficient (ob/ob) mouse. We observed a significant increase in the total lipid concentration in obese EVs-iWAT. Principal component analysis (PCA) of lipidomic datasets revealed a distinct separation between WT EVs-iWAT and ob/ob EVs-iWAT (Figure 3A, Supplementary Figure S3A), highlighting substantial lipidomic changes in the ob/ob model. Prominent increases in several lipid species, including SPHP, StE, TG, FA, PE, ST, PIP, PIP2, PIP3, OAHFA, PG, LPC, LPE, and LPG were decreased in obese EVs-iWAT compared with the lean controls (Figure 3B, Supplementary Figure S3B). Then, we analyzed the subclasses based on the values of predictive variable importance (VIP) obtained from the OPLS-DA model for VIP> 1, *p* < 0.05 as differential lipid molecules visualized using bubble plots. The results also confirmed the significant upregulation of several TG, PE, and ST species and the downregulation of PC and CL species in obese EVs-iWAT (Figure 3C; Supplementary Table S4). Furthermore, we observed a significant decrease of carbon atoms in the acyl chain composition of several lipid classes, including CL, CerG3NAc2, and GD2, and an increase of carbon atoms in FA with 20 carbon atoms and Hex2Cer with 36–42 carbon atoms (Supplementary Figure S3C).

We also observed notable lipidomic changes in EVs-eWAT in the ob/ob model (Figure 3D, Supplementary Figure S4A). Several lipid species, including FA, PC, PE, DG, WE and SM, were significantly increased, while PIP, PIP2, and PIP3 were decreased in obese EVs-eWAT. Interestingly, LPC and LPE, which were decreased in obese EVs-iWAT, exhibited an increase in obese EVs-eWAT (Figure 3E, Supplementary Figure S4B). The PC/PE ratio, inversely related to insulin sensitivity in adipocytes, decreased in obese EVs-eWAT but increased in obese EVs-iWAT. Further VIP lipid analysis reconfirmed the significant upregulation of several PC, PE, DG, Hex2Cer and downregulation of CerP, Cer, and OAHFA species in obese EVs-eWAT (Figure 3F, Supplementary Table S5). Furthermore, we observed a significant decrease of carbon atoms in the acyl chain composition of CO, MG, and DG, while carbon atoms of several species of LPC, LPG, and OAHFA decreased in obese EVs-eWAT (Supplementary Figure S4C).

In the EVs-BAT, PCA analysis also revealed substantial lipidomics changes in the ob/ob model (Figure 3G, Supplementary Figure S5A). Interestingly, despite the significant increase in total lipid concentration in obese EVs-eWAT, the lipidome profile in obese EVs-BAT tended to shift towards a more EVs-WAT-like composition (Figure 3H, Supplementary Figure S5B). Several lipid species, including TG, FA, PC, PE, LPE, LPG, and SM were significantly upregulated, while PIP2 and PG were downregulated in obese EVs-BAT. Furthermore, VIP lipid analysis confirmed the significant upregulation of several PC, PE, and SM species and the downregulation of PG and OAHFA species in obese EVs-BAT (Figure 3I; Supplementary Table S6). Furthermore, unlike EVs-iWAT and EVs-eWAT, carbon numbers of several lipids, including Hex1Cer, CL, Cer, FA, and PS, were significantly increased in obese EVs-BAT (Supplementary Figure S5C). These results underscore the profound impact of obesity on the composition, structure, and lipidomic profiles of EVs-AT.

### 3.4 Function analysis identifies altered lipid expression involved in metabolism and inflammation in obese EVs-AT

**FIGURE 4:**
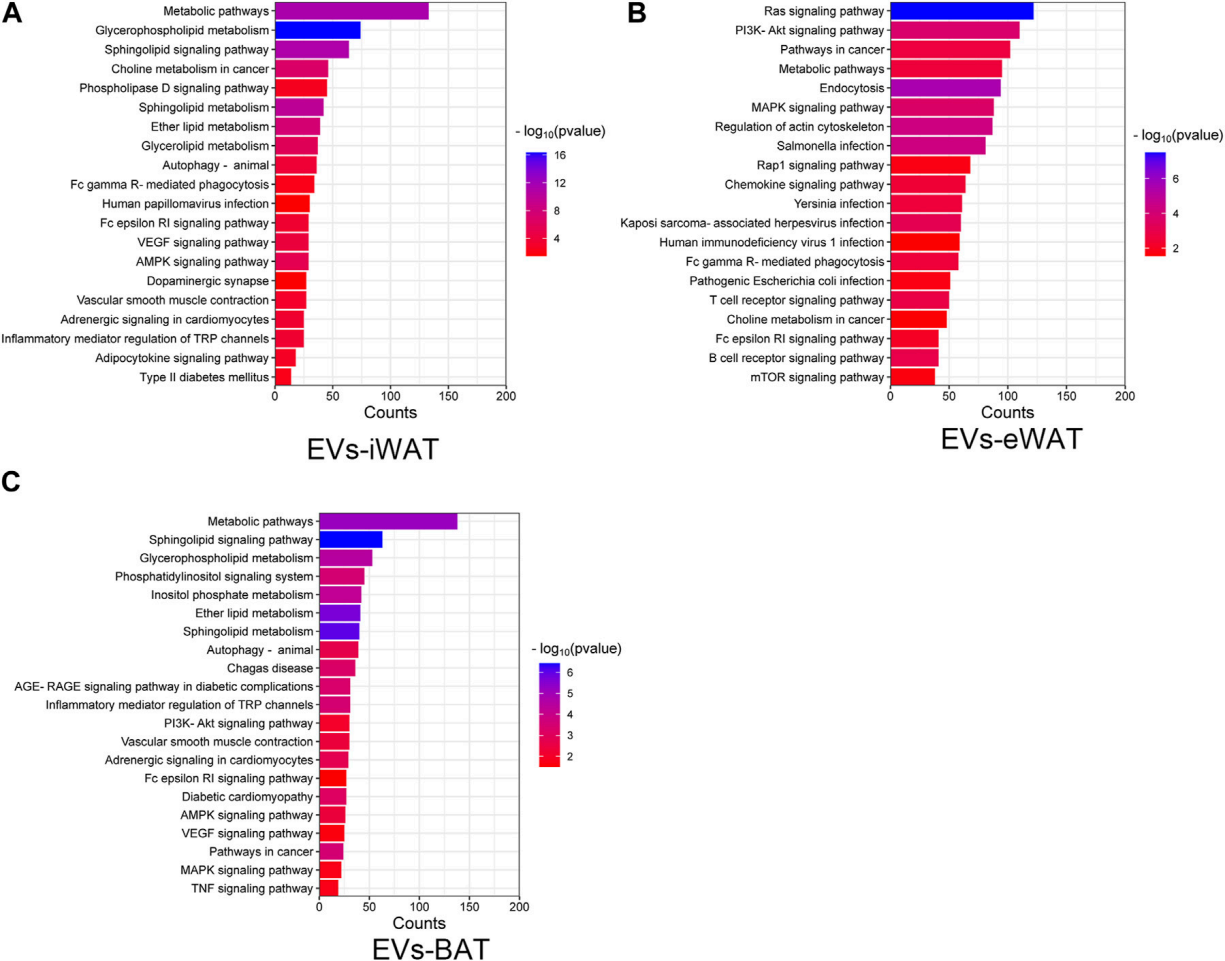
KEGG pathway analysis of the changed lipids in obese EVs-iWAT **(A)**, EVs-eWAT **(B)** and EVs-BAT **(C)**.

We performed an enrichment analysis to elucidate the functional implications of the differentially expressed lipids in obese EVs-AT. In EVs-iWAT, the differential lipids were associated with metabolic pathways, glycerophospholipid metabolism, sphingolipid signaling pathway, and choline metabolism. Additionally, pathways related to type II diabetes mellitus, adipocytokine signaling pathway, and adrenergic signaling in cardiomyocytes were also involved, indicating an association of the upregulated lipids with endocrine regulation (Figure 4A). Further analysis revealed an enrichment of the B cell receptor signaling pathway, T cell receptor signaling pathway, chemokine signaling pathway and PI3K-Akt signaling pathway in EVs-eWAT, indicating an association of the upregulated lipids with inflammation regulation, which was in contrast with EVs-iWAT (Figure 4B). In EVs-BAT, we found that altered lipids were involved in inflammatory mediator regulation of TRP channels, PI3K-Akt signaling, and TNF signaling pathways (Figure 4C).

**FIGURE 5:**
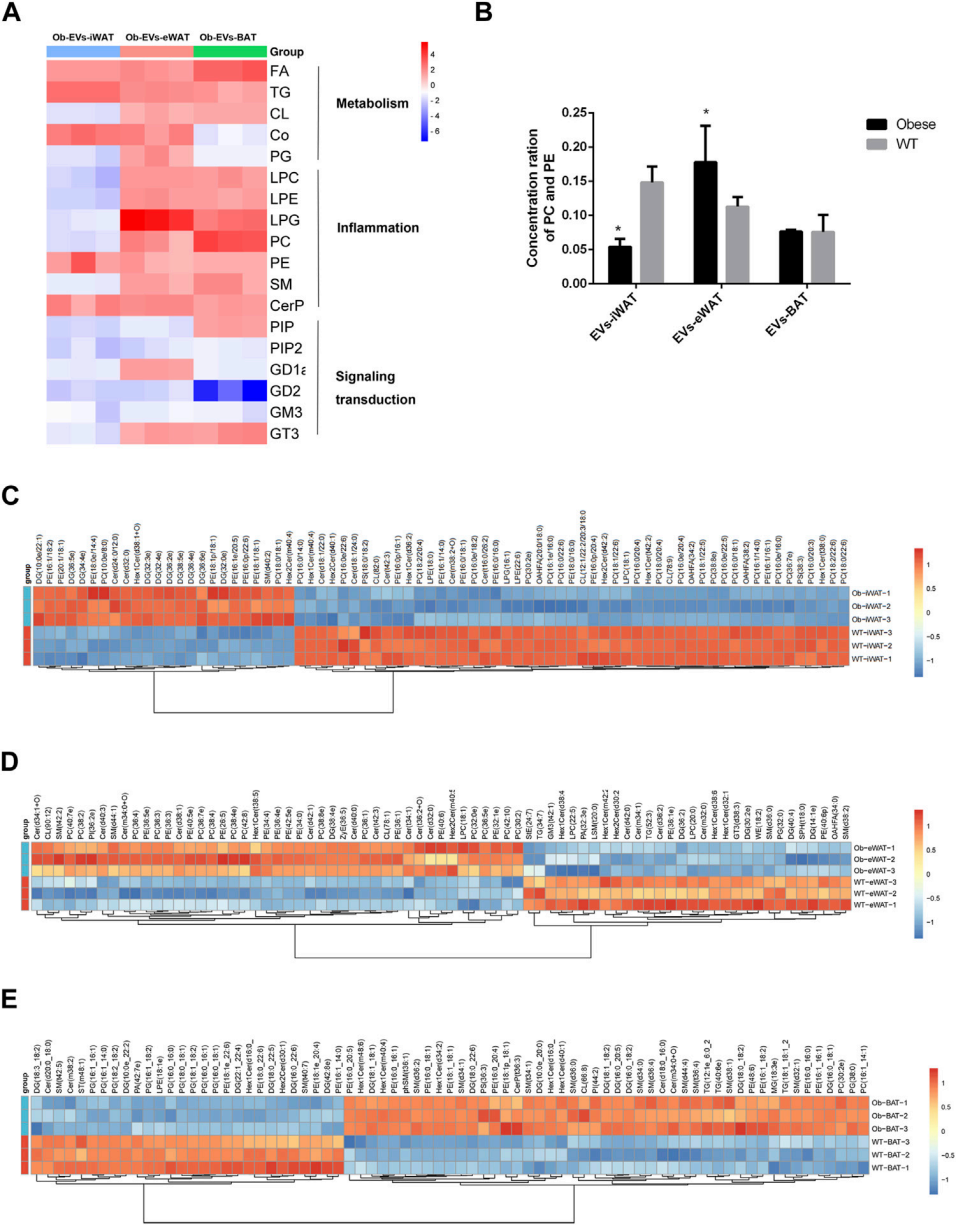
Functional analysis of the changed lipids in obese EVs-AT. **(A)** A heatmap of changed lipid classes in obese EVs-AT, the fold change of lipid classes compared to the lean EVs-AT was represented by color ranges from blue to red, as indicated by the scale bar; **(B)** The concentration of PC and PE in the lean and obese EVs-iWAT, EVs-eWAT and EVs-BAT; **(C)** Hierarchical clustered heatmaps of lipid species in the lean and obese EVs-iWAT; **(D)** Hierarchical clustered heatmaps of lipid species in the lean and obese EVs-eWAT; **(E)** Hierarchical clustered heatmaps of lipid species in the lean and obese EVs-BAT.

We further analyzed the functions of differentially expressed lipid classes in obese EVs-AT, summarizing their involvement in the regulation of cell metabolism, inflammation, and signaling transduction. In obese EVs-iWAT, we observed an increase in lipids associated with metabolism, such as FA, TG, and Co, while most lipids involved in inflammation (LPC, LPE, PC, PE, SM, CerP) and signaling transduction (PIP, PIP2, GD1a, GD2, GM3) were decreased (Figure 5A). Moreover, the PC/PE ratio, a contributor to the development of insulin resistance, was increased (Figure 5B). We also used multivariate statistical analysis to identify the top 75 differential lipid substances, revealing 20 PC species, 7 PE species, 2 LPE species, and 1 LPC species to be downregulated in obese EVs-iWAT (Figure 5C).

In contrast to obese EVs-iWAT, obese EVs-eWAT exhibited a significant increase in lipids associated with inflammation, accompanied by a decrease in lipids related to signaling transduction (Figure 5A). The PC/PE ratio was significantly increased in obese EVs-eWAT, indicating a potential development of insulin resistance (Figure 5B). Furthermore, analysis of lipid substances revealed significant upregulation of 14 PC species, 11 PE species, and 10 Cer species. Furthermore, the other inflammation-related lipids, including 3 LPC species and 1 LPE species, were also upregulated (Figure 5D), collectively suggesting that obese EVs-eWAT may contribute to the development of low inflammation states in obesity.

Similar to obese EVs-eWAT, inflammation-associated lipids were significantly increased, while those linked to signal transduction decreased in obese EVs-BAT (Figure 5A). Analysis of lipid substances showed that 10 PG species were significantly decreased (Figure 5F). Since PG is predominantly located in mitochondrial and microsomal membranes, this decrease may indicate a decrease of mitochondria in EVs or an impaired metabolic homeostasis regulation. Furthermore, we also noted an increase in several inflammation-related lipids, including 9 SM species. These findings indicate that lipid species enriched in obese EVs-AT could serve as potential biomarkers or mediators of obesity-associated metabolic dysfunctions.

By generating chord plots, we can clearly observe the differences among the obese EVs-iWAT (Supplementary Figure S6A) and EVs-eWAT (Supplementary Figure S6B) and EVs-BAT (Supplementary Figure S6C). Our results suggested that three groups presented similar lipid correlation patterns. However, the three groups also had substantial patterned differences. For instance, the positive correlations between PC and PE are stronger in EVs-eWAT than EVs-iWAT and EVs-BAT. On the contrary, the positive correlations between PG and DG are stronger in EVs-iWAT and EVs-BAT than EVs-eWAT. Therefore, lipidomic correlation patterns provided more comprehensive molecular insights into assessing the influence of obesity on the regulation of metabolism and inflammation of EVs-AT.

## 4 Discussion

Previous studies highlighted the significance of EVs-AT as critical mediators of metabolic alterations associated with obesity-related diseases (Zhang et al., 2016; Gesmundo et al., 2021; Zhang et al., 2021). However, limited studies have focused on the role of EVs-AT lipids in the development of obesity-related diseases. Our study provides comprehensive lipid maps of EVs derived from subcutaneous WAT, visceral WAT, and BAT, revealing the alterations of specific lipid classes or species within obese EVs-AT, which may contribute to the pathogenesis of obesity-related diseases.

We found that SPH, PE, DG, TG, ZyE, Cer, and SM were the commonly enriched lipids in EVs-iWAT, EVs-eWAT, and EVs-BAT. Notably, enrichment of ZyE, Cer, and SM have been reported in other EV types, which might provide a higher EV membrane order degree and contribute to increasing the rigidity of EVs and increase EV resistance to non-ionic detergents. (Skotland et al., 2020; Su et al., 2021; Blandin et al., 2023). Moreover, we found that SPH, PE, DG, and TG were specifically enriched in EVs-AT compared to EVs derived from other tissues. The accumulation of TG and DG (associated with TG turnover) in EVs-AT indicates that EVs-AT could provide energy to recipient cells and regulate energy metabolism. Furthermore, both sphingosines (SPH) and PE play an important role in various complex biological processes such as signal transduction, endocytosis, cell metabolism, and apoptosis (van der Veen et al., 2017; Ogretmen, 2018), indicating that EVs-AT may facilitate vital cellular communication processes.

Interestingly, we observed distinct differences in lipid class composition between EVs-iWAT, EVs-eWAT, and EVs-BAT. For instance, SM, LPE, LPC, and LPG, which were closely related to inflammation, were enriched in EVs-eWAT (Zhang et al., 2018). This indicates that EVs-eWAT may mediate the regulation of inflammation through lipid transfer. However, PG, primarily located in mitochondrial and microsomal membranes, was enriched in EVs-BAT compared to the white adipose tissue-derived EVs (EVs-iWAT and EVs-eWAT) (Morita and Terada, 2015). Previous studies have reported that PG can be incorporated into cardiolipin to improve mitochondrial activity and inhibit inflammation (Chen et al., 2018). Therefore, EVs-BAT may transfer mitochondria and regulate mitochondrial activity. These results suggest that EVs-eWAT might be more active in the regulation of inflammation, while EVs-BAT might be involved in thermogenic and metabolic activities from the basal lipidomic standpoint.

Our investigation revealed that obesity-induced significant changes in the lipid profiles of EVs-AT. Firstly, we noticed an overall increase of longer lipids species in obese EVs-AT, especially in EVs-BAT. Recent studies have established that a change in acyl chain length affects the pathogenesis of various human diseases, including cancer, cardiomyopathy, and metabolic diseases (Grösch et al., 2012; Park and Park, 2015). For instance, the long-chain ceramides were increased in irritable bowel syndrome and cystic fibrosis, where it induces apoptosis (Grösch et al., 2012). Consistently, we also found an increase in the long-chain ceramides in obese EVs-eWAT and EVs-BAT. Although the chain length of the other lipids was also increased, limited studies have focused on their pathological roles in the adipose tissue or the EVs-AT. Understanding the significance of such changes in obesity could potentially provide novel markers for the diagnosis of obesity-related disease.

Moreover, we observed a marked decrease in lipid classes associated with signal transduction in obese EVs-AT, indicating impaired biological functions. Specifically, PIP2, which plays a crucial role in regulating many biological processes such as signaling transduction, intracellular trafficking, membrane dynamics, and cell-matrix adhesion, was decreased in obese EVs-iWAT and EVs-BAT compared to obese EVs-eWAT (Mandal, 2020). PIP2 is central to these processes. PIP2 binds to protein kinase AKT/PKB and is implicated in the PIP3-Akt signaling pathway (Shen et al., 2022). The PI3K/AKT signaling pathway promotes lipid biosynthesis and inhibits lipolysis in adipose tissue and liver (Li et al., 2011; Huang et al., 2018). During obesity, inactivation of the PI3K/AKT signaling pathway can inhibit lipolysis, leading to impaired glucose utilization (On et al., 2008; Yue and Lam, 2012). This process triggers the ectopic accumulation of lipids and subsequently causes insulin resistance. Moreover, decreased activation of the PI3K/AKT pathway can also reduce insulin secretion and impair β cell function (Huang et al., 2018), further aggravating insulin resistance in multiple tissues. These findings indicated that, in addition to miRNAs and adipokines, lipids within EVs-AT also mediate the development of insulin resistance (Kahn et al., 2019).

Furthermore, our study suggests that lipids may mediate EVs-AT-induced inflammation in obesity. Several glycerophospholipids were significantly increased in obese EVs-eWAT and EVs-BAT. Previous studies have shown the correlation between glycerophospholipids and inflammation, particularly in obesity (Bansal et al., 2016; Darabi and Kontush, 2016). For instance, LPC serves as a pro-inflammatory lipid and enhances the release of interleukin-1β (IL-1β), IL-6, and tumor necrosis factor-α (TNF-α) from adipocytes, enhances secretion of interferon-γ from peripheral blood mononuclear leucocytes, and increases activation of B cells (Darabi and Kontush, 2016; Liu et al., 2020). Further studies have also demonstrated an increased PC expression in obese intestinal epithelial cells. The EVs-derived PC binds to and activates AhR (Aryl Hydrocarbon Receptor), subsequently inhibiting the expression of genes essential for activation of the PI3K/AKT signaling pathway (Kumar et al., 2021). Consequently, this can negatively affect insulin signaling when the EVs are taken up by macrophages and hepatocytes, leading to inhibition of the insulin signaling pathway. We also observed a decrease in the PC/PE ratio in EVs-iWAT and EVs-BAT. Previous studies have indicated that this is critical for insulin signaling, with a reduced PC/PE ratio improving insulin signaling in hepatocytes (van der Veen et al., 2017; van der Veen et al., 2019). This indicates that the ratio of PC and PE in EVs-AT could serve as a potential marker for insulin resistance, and obese EVs-AT may alter the PC/PE ratio of targeted cells, including the adipocytes and liver cells, thereby mediating the development of insulin resistance in obesity. In recent years, there has been increased awareness of the importance of adipose tissue macrophages in obesity. As a response to specific microenvironmental stimuli, levels of proinflammatory M1-like ATM subtypes are elevated, whereas antiinflammatory M2-like macrophages are reduced. The M1-like ATMs have been shown to impair insulin signaling in both mice and humans. Lipids regulate signal transduction and gene regulation during macrophage activation. Dysregulated lipid metabolism is implicated in aberrant macrophage functions in obesity. Macrophage lipid synthesis regulates bioenergetics, phagocytosis, and inflammatory cytokine production. Considering that macrophages were important EV providers and enriched in obese AT. Therefore, we speculate that macrophage-derived EVs could be involved in obesity-associated EVs-AT lipid fingerprints. In summary, our study highlights the role of lipids in obese AT-EVs as inflammatory mediators, a major contributor to decreased insulin sensitivity.

## 5 Conclusion

In summary, the current study presented a comprehensive analysis of lipidome in EVs derived three different types of adipose tissue on scale of the entire lipidome analysis, providing new insight in comprehensive understanding of the heterogeneity of EVs-AT. In this study, we reported that the lipids of obese EVs-AT might also correlated with lipogenesis, inflammatory response and insulin resistance. This study implied that EVs-AT could be served as a novel carrier for lipokines and mediated the biological functions of EVs-AT. However, further research is needed to explore the lipid profiles in the lean/obese human EVs dervied from iWAT, eWAT and BAT and verified their physiological or the pathological roles. Taken together, our work will facilitate identifying new therapeutic targets and developing new strategies to combat metabolic diseases.

## Data Availability

The original contributions presented in the study are included in the article/Supplementary Material, further inquiries can be directed to the corresponding author.
